# Preoperative Hill grade versus hiatal hernia size: which drives the decision for Dor fundoplication during sleeve gastrectomy? A retrospective cohort study

**DOI:** 10.3389/fsurg.2026.1934674

**Published:** 2026-09-03

**Authors:** Xing Du, Hongyi Dong, Diangang Liu

**Affiliations:** 1 Department of General Surgery, Xuan Wu Hospital, Capital Medical University, Beijing, China; 2 Capital Medical University, Beijing, China

**Keywords:** dor fundoplication, gastroesophageal reflux disease, hiatal hernia, hill grade, propensity score overlap weighting, sleeve gastrectomy

## Abstract

**Background:**

Reflux risk after sleeve gastrectomy is closely linked to gastroesophageal junction anatomy, but the relative influence of preoperative Hill grade vs. hiatal hernia size on the decision to add concurrent Dor fundoplication remains unclear. Identifying the dominant driver may improve patient selection for an adjunctive anti-reflux procedure.

**Objective:**

To compare the relative contributions of Hill grade and hiatal hernia size to the decision for Dor fundoplication during sleeve gastrectomy, and to evaluate whether Dor fundoplication is associated with improved reflux outcomes at 12 months.

**Methods:**

This single-centre retrospective cohort study included adult patients who underwent primary sleeve gastrectomy between January 2023 and June 2025. Follow-up was completed and the database locked in June 2026. Patients were divided into Dor and non-Dor groups according to whether concurrent Dor fundoplication was performed. The primary analyses examined factors associated with Dor selection and treatment failure for reflux at 12 months. Secondary outcomes included *de novo* GERD, persistent GERD, PPI discontinuation, endoscopic and pH-monitoring evidence of reflux, safety, weight loss, and diabetes remission. To minimize selection bias inherent in surgeon-driven treatment decisions, we applied propensity score overlap weighting to create a pseudo-population with balanced baseline covariates between groups, simulating the conditions of a randomized trial. Multivariable logistic regression, sequential model comparisons, decision curve analysis, and propensity score overlap weighting were used to control confounding.

**Results:**

Among 316 patients (126 Dor, 190 non-Dor), high-risk Hill grade was the strongest independent factor for Dor selection (adjusted OR 3.48, 95% CI 2.02–6.00). Hiatal hernia size showed a weaker association (adjusted OR 1.42 per 1-cm increase, 95% CI 1.10–1.85). Model comparisons confirmed a greater incremental contribution from Hill grade than from hernia size. After overlap weighting, Dor was associated with a lower risk of treatment failure for reflux (adjusted RR 0.63, 95% CI 0.43–0.91), with consistent reductions in *de novo* GERD, persistent GERD, and regular PPI use, and a higher PPI discontinuation rate. Operative time was longer in the Dor group, but hospital stay, major complications, weight loss, and diabetes remission did not differ significantly. Sensitivity analyses supported the main findings.

**Conclusion:**

In patients undergoing sleeve gastrectomy, preoperative Hill grade drives the decision for concurrent Dor fundoplication more strongly than hiatal hernia size. After adjustment for clinical comparability, Dor fundoplication was associated with a lower risk of treatment failure for reflux at 12 months, without an apparent safety or weight-loss penalty.

## 1 Introduction

Obesity has become one of the fastest-growing chronic disease burdens worldwide. A global pooled analysis based on data from 222 million individuals between 1990 and 2022 showed that by 2022, more than one billion people were living with obesity, with adult obesity rates having risen markedly since 1990 and even greater increases seen among children and adolescents (1). Recent GBD forecasting studies suggest that if past trends continue, the number of overweight and obese adults could reach 3.8 billion by 2050, and the burden of obesity-related metabolic, cardiovascular, and digestive diseases will continue to grow (2). Against this background, metabolic and bariatric surgery has evolved from a purely weight-loss intervention to an important treatment for obesity-related metabolic diseases. The 2022 ASMBS/IFSO guidelines further expanded the indications for metabolic and bariatric surgery (3). Sleeve gastrectomy has become the most commonly performed primary metabolic and bariatric procedure worldwide, owing to its relative technical simplicity, stable weight-loss effects, and limited impact on nutritional absorption (4). However, as the volume of procedures has increased, preoperative risk assessment of gastroesophageal reflux disease and postoperative reflux management have gradually become key issues in quality control for sleeve gastrectomy. The IFSO has also emphasised the clinical value of perioperative upper gastrointestinal endoscopy in metabolic and bariatric surgery (5).

Sleeve gastrectomy provides sustained weight loss and metabolic benefits, but its effect on gastroesophageal reflux disease remains controversial. Long-term randomised trials have shown that both sleeve gastrectomy and Roux-en-Y gastric bypass achieve stable weight loss, but reflux oesophagitis or *de novo* or worsening GERD is more common after sleeve gastrectomy (6–8). This phenomenon may be related to reduced gastric fundus compliance, changes in the angle of His, impairment of the anti-reflux barrier at the gastroesophageal junction, increased pressure within the gastric sleeve, and anatomical abnormalities in the hiatal region (9). Postoperative GERD should therefore not be regarded merely as a symptom issue, but rather as the result of interplay among gastric sleeve morphology, hiatal structure, and gastroesophageal junction function (10). Systematic reviews suggest that the incidence of Barrett's oesophagus, reflux oesophagitis, PPI use, and hiatal hernia may increase after sleeve gastrectomy (11). Previous studies have also shown that symptoms do not always correlate with endoscopic findings, and relying solely on symptoms may underestimate the objective reflux burden (12). The IFSO Barrett's Oesophagus Working Group further emphasised that Barrett's oesophagus is closely related to the choice of metabolic and bariatric procedure, and that both preoperative evaluation and long-term follow-up should take this into account (13).

Identifying patients at high risk of reflux before surgery is the key to deciding whether an adjunctive anti-reflux procedure is needed. The ASMBS position statement on upper gastrointestinal endoscopy states that endoscopy can detect oesophagitis, Barrett's oesophagus, hiatal hernia, and other lesions that may affect procedure selection (14). The ACG guidelines emphasise that pH monitoring, impedance monitoring, and oesophageal manometry can provide objective evidence for GERD diagnosis and treatment decisions (15). The AGA recommendations also note that GERD evaluation should integrate symptoms, endoscopy, reflux monitoring, and patient characteristics, and that reflux risk should be fully assessed when selecting sleeve gastrectomy in patients with obesity (16). Among commonly used preoperative indicators, Hill grade, assessed by retroflexed endoscopic view of the gastroesophageal flap valve, reflects the integrity of the anti-reflux barrier at the gastroesophageal junction. Recent studies have shown that Hill grade is associated with age, hiatal hernia, lower oesophageal sphincter pressure, acid reflux, and severity of reflux oesophagitis (17). In the sleeve gastrectomy population, preoperative Hill grade III-IV has been shown to be independently associated with postoperative GERD and may help guide procedure selection (18). Another study also suggested that Hill grade can predict the risk of GERD and reflux oesophagitis after sleeve gastrectomy (19). Hiatal hernia size mainly reflects the degree of anatomical defect in the hiatal region. Systematic reviews have shown that sleeve gastrectomy combined with hiatal hernia repair or with fundoplication may improve reflux outcomes, but the appropriate populations and risk–benefit profiles of different strategies are not the same (20). Recent studies suggest that concurrent hiatal hernia repair may improve reflux symptoms in some patients and reduce *de novo* GERD, but whether it can replace reconstruction of the anti-reflux barrier remains unclear (21).

Against this background, concurrent fundoplication during sleeve gastrectomy has been proposed as a modified strategy that balances weight loss and reflux control. A systematic review showed that fundoplication sleeve gastrectomy achieves weight-loss outcomes similar to those of conventional sleeve gastrectomy and may reduce the incidence of postoperative GERD (22). Among these, the Sleeve-Dor technique, which uses an anterior partial fundoplication to reinforce the anti-reflux barrier at the gastroesophageal junction, has been reported as a feasible procedure, especially for obese patients with preoperative reflux risk (23). The D-SLEEVE study also suggested that sleeve gastrectomy combined with anterior fundoplication can improve or prevent mild-to-moderate GERD while preserving the restrictive weight-loss effect (24). Five-year follow-up data from Nissen sleeve gastrectomy showed that combined fundoplication may provide good long-term reflux control, although the completeness of endoscopic follow-up and standardisation of the technique require further validation (25). A recent systematic review also pointed out that although fundoplication sleeve gastrectomy may improve reflux outcomes, it may also increase complications and reoperation rates. Therefore, this procedure should not be used routinely, but should be selected on the basis of preoperative reflux risk (26).

Existing studies have largely focused on the risk of GERD after sleeve gastrectomy, or have compared outcomes of pure sleeve gastrectomy, concurrent hiatal hernia repair, and concurrent fundoplication. Previous investigations have separately linked Hill grade (17–19) and hiatal hernia size (20, 21) to postoperative GERD risk. Earlier work focused on predicting outcomes, such as which patients would develop postoperative GERD. However, a more direct question in clinical decision-making remains unanswered: when deciding whether to add a Dor fundoplication, do surgeons rely mainly on the functional abnormality of the gastroesophageal flap valve reflected by Hill grade, or on the anatomical defect of the hiatus reflected by hiatal hernia size? At present, quantitative evidence on the relative influence of these two factors on the choice of Dor fundoplication is lacking. Clarifying this question would help us understand the real decision-making logic behind adjunctive anti-reflux procedures during sleeve gastrectomy and would support the development of a clearer preoperative risk-stratification framework. This finding extends the existing literature in two important ways. First, it identifies Hill grade as the dominant driver of surgical decision-making, providing a clearer target for preoperative risk stratification than hernia size alone. Second, it supports the growing body of evidence that fundoplication sleeve gastrectomy can improve reflux outcomes without compromising weight loss (22, 24), but adds the critical nuance that patient selection should prioritise functional assessment of the gastroesophageal junction over anatomical measurement alone.

Therefore, this study aimed to compare the relative influence of preoperative Hill grade and hiatal hernia size on the selection of concurrent Dor fundoplication in patients undergoing primary sleeve gastrectomy, and to further evaluate the association of Dor fundoplication with reflux-related outcomes, safety, and weight-loss and metabolic outcomes at 12 months postoperatively. The goal was to connect preoperative anatomical risk, surgical decision-making, and postoperative reflux outcomes, and to provide a clearer clinical basis for the individualised selection of adjunctive anti-reflux procedures during sleeve gastrectomy.

## 2 Methods

### 2.1 Study design, data source, and ethics

This was a single-centre retrospective cohort study that consecutively included adult patients with obesity who underwent primary laparoscopic or robot-assisted sleeve gastrectomy at XuanWu Hospital affiliated to Capital Medical University between 1 January 2023 and 30 June 2025. The study population was derived from a prospectively maintained surgical registry database of the metabolic and bariatric surgery centre, and data were cross-verified using the hospital electronic medical record system, endoscopy database, surgical anaesthesia records, discharge summaries, outpatient follow-up records, prescription systems, and postoperative gastroscopy reports.

This study did not use a historical control design. Both the Dor fundoplication group and the non-Dor group came from patients who underwent sleeve gastrectomy within the same study time window. Group assignment was based on whether concurrent Dor fundoplication was performed during the index procedure, not on surgical year, changes in clinical pathways, or perioperative management phases. Surgical year, surgeon identity, and surgical approach were treated as potential confounders in the statistical analyses.

Follow-up data were collected up to 30 June 2026, and the database was locked on the same date. Because this study used 12-month reflux-related outcomes as key clinical endpoints, the last date of inclusion was set at 30 June 2025. This timeline ensured that the last enrolled patient still had the opportunity to complete at least 12 months of follow-up and avoided time-related bias due to insufficient follow-up windows.

The study protocol was reviewed and approved by the Ethics Committee of Xuan Wu Hospital, Capital Medical University [(2025) 288-001]. This was a retrospective observational study; all surgical strategies were determined by the clinical team during routine care on the basis of patient symptoms, endoscopic findings, hiatal anatomical status, intraoperative findings, and surgeon judgment. The research team did not assign patients to study groups, did not alter the established treatment plans, and did not perform any additional research interventions.

Because this study used only clinical data generated during routine care, and all data were de-identified before analysis, the ethics committee granted a waiver of written informed consent. The study was conducted in accordance with the Declaration of Helsinki and the institution's clinical data management requirements.

### 2.2 Patient selection

#### 2.2.1 Inclusion criteria

Patients had to meet all of the following criteria: age ≥18 years; met the indications for metabolic and bariatric surgery; underwent primary laparoscopic or robot-assisted sleeve gastrectomy; completed upper gastrointestinal endoscopy within 12 months before surgery; Hill grade recorded in the endoscopy report or available on reviewable images; hiatal hernia status or hernia size available from preoperative endoscopy, upper gastrointestinal contrast study, or operative records; and completed at least 12 months of follow-up, or had a clear censoring time and reason.

#### 2.2.2 Exclusion criteria

Patients were excluded if they met any of the following: previous metabolic and bariatric surgery or revisional bariatric surgery; concurrent Roux-en-Y gastric bypass, one-anastomosis gastric bypass, or other primary procedure other than sleeve gastrectomy; preoperative Barrett's oesophagus with dysplasia; preoperative Los Angeles class C or D reflux oesophagitis; large hiatal hernia, paraoesophageal hernia, or hiatal hernia diameter >5 cm; achalasia, absent oesophageal contractility, or severe oesophageal motility disorder; surgery during pregnancy; gastric resection for malignancy, gastric pathology, or other non-obesity-related reasons; missing core variables or primary outcomes that could not be confirmed after medical record review.

#### 2.2.3 Screening process

During the study period, 412 adult patients who underwent primary sleeve gastrectomy or sleeve-related procedures were screened. According to the inclusion and exclusion criteria, 96 patients were excluded. Among them, 31 lacked preoperative endoscopy within 12 months or had no Hill grade recorded; 12 had no confirmable hiatal hernia status or size; 15 were revisional cases after previous bariatric surgery; 8 had Barrett's oesophagus with dysplasia or Los Angeles class C/D reflux oesophagitis; 10 had large hiatal hernia, paraoesophageal hernia, or hernia diameter >5 cm; 5 had severe oesophageal motility disorders; 3 underwent gastric surgery for pregnancy, malignancy, or other non-obesity-related reasons; and 12 did not complete 12 months of follow-up with unconfirmed primary outcomes.

Finally, 316 patients were included in the analysis. Among them, 126 received concurrent Dor fundoplication during sleeve gastrectomy and were assigned to the Dor group, while 190 underwent sleeve gastrectomy without Dor fundoplication and were assigned to the non-Dor group.

### 2.3 Surgical strategy and grouping

#### 2.3.1 Sleeve gastrectomy

All operations were performed by senior surgeons with metabolic and bariatric surgical qualifications or by a fixed surgical team under their supervision. Patients were placed in the supine split-leg position, and after pneumoperitoneum was established, the procedure was completed using a laparoscopic or robot-assisted approach. During the operation, the abdominal cavity was first explored to confirm the anatomy of the gastroesophageal junction, the angle of His, the greater curvature of the stomach, and the hiatal region. The omental vessels were then divided along the greater curvature starting approximately 4–6 cm from the pylorus, and dissection proceeded proximally to the angle of His, with full exposure of the left crus, the posterior gastric fundus, and the area around the gastroesophageal junction. The short gastric vessels and posterior fundic adhesions were completely managed to avoid retained fundic pouches, gastric sleeve twisting, or proximal stenosis.

All sleeve formations were performed with a 36–40 Fr bougie as a guide. The bougie was placed by the anaesthetist and advanced along the lesser curvature through the antrum and body to the gastroesophageal junction to ensure a consistent sleeve diameter. The stapler was fired sequentially from distal to proximal along the lateral aspect of the bougie; the distal transection line was designed to preserve antral pump function, and the proximal transection line was placed close to the lateral aspect of the angle of His. Staple cartridge selection was based on gastric wall thickness and manufacturer recommendations, with staple-line reinforcement used when necessary. After completion of the sleeve, the surgeon routinely inspected the sleeve morphology, staple-line integrity, orientation of the gastroesophageal junction, and any obvious twisting or stenosis. If an intraoperative leak test was performed, it was done using methylene blue, air insufflation, or endoscopic assistance, according to centre routine and surgeon preference.

In the non-Dor group, the fundic tissue was resected as completely as possible according to standard sleeve gastrectomy principles, to minimise the potential impact of retained fundus on postoperative reflux and weight outcomes. In the Dor group, when creating the proximal sleeve, the surgeon preserved a small portion of seromuscular tissue or a small fundic patch adjacent to the gastroesophageal junction that could be used for anterior partial fundoplication. The extent of preserved tissue was limited to that needed for a tension-free covering of the distal oesophagus without compromising sleeve patency. The remainder of the fundus was resected according to standard sleeve principles. In all patients, the longitudinal axis of the sleeve was confirmed to be straight, the bougie passed smoothly, and the gastroesophageal junction was free from obvious angulation before the end of the procedure.

#### 2.3.2 Dor fundoplication

Dor fundoplication was defined only as anterior partial fundoplication performed concurrently during the index sleeve gastrectomy. This procedure was carried out after completion of the sleeve and confirmation that the sleeve was not significantly narrowed or twisted. The surgeon again exposed the distal oesophagus, the gastroesophageal junction, and both crura, confirming that the preserved tissue had sufficient mobility to cover the anterior wall of the distal oesophagus without tension. If a hiatal hernia or hiatal laxity was present, the hiatal region was managed before Dor fundoplication.

The Dor fundoplication was performed according to a standardised institutional protocol as described below, which was uniformly adopted by all participating surgeons to minimise technical variability. The decision to perform Dor fundoplication was based on the same preoperative and intraoperative criteria for all surgeons, including Hill grade, hernia size, reflux symptoms, and intraoperative assessment of the gastroesophageal junction. For construction of the Dor fundoplication, the preserved tissue adjacent to the gastroesophageal junction or the small fundic patch was rotated anteriorly to cover the distal oesophagus and the anterior wall of the gastroesophageal junction over approximately 180°. The length of the fundoplication usually covered 2–3 cm of the distal oesophagus, limited to avoid compression of the lower oesophagus, proximal narrowing of the sleeve, or angulation at the gastroesophageal junction. The fundoplication was secured with interrupted 2–0 or 3–0 non-absorbable or long-acting absorbable sutures. Sutures were placed through the seromuscular layer or the superficial muscle layer of the oesophagus, avoiding mucosal penetration. Fixation points typically included the folded tissue to the anterior wall of the distal oesophagus, the seromuscular layer of the gastroesophageal junction, and the perihiatal tissues. After completion, the surgeon confirmed that the wrap was tension-free, not overly encompassing, and not significantly narrowed, with a patent sleeve lumen, using the bougie or endoscopic assistance. Key technical requirements for Dor fundoplication were anterior partial coverage, low-tension fixation, and avoidance of circumferential compression. Patients who underwent repeat anti-reflux surgery for reflux, stenosis, or other reasons after the index procedure were not reclassified as Dor-exposed at the index operation.

#### 2.3.3 Hiatal hernia repair

The hiatal region, the angle of His, and the position of the gastroesophageal junction were routinely inspected during surgery. If preoperative studies or intraoperative findings suggested a hiatal hernia, upward displacement of the gastroesophageal junction, crura laxity, or insufficient intra-abdominal oesophageal length, the phrenoesophageal ligament was further dissected and both crura were exposed. The hernia sac was reduced, and the distal oesophagus was mobilised into the mediastinum as needed until the gastroesophageal junction lay below the diaphragm without tension and an adequate intra-abdominal oesophageal length was obtained. Care was taken to preserve the vagus nerves and adjacent vascular structures during dissection.

Hiatal hernia repair aimed to restore anatomical closure of the crura and a subdiaphragmatic position of the gastroesophageal junction. Repair was usually performed with interrupted posterior crural sutures, typically using 0 or 2–0 non-absorbable sutures. Sutures were placed behind the oesophagus to approximate the right and left crura until the hiatus accommodated the oesophagus and the bougie without obvious compression. If anterior hiatal laxity remained after posterior repair, additional anterior reinforcing sutures could be placed according to intraoperative findings. Mesh was not used routinely; it was used only at the surgeon's discretion when the crural tissue was weak, primary closure was under high tension, or the hiatal defect was large but still within the inclusion criteria of this study. Hiatal hernia repair was recorded as an independent surgical procedure and was not equivalent to Dor fundoplication, nor was it used as a criterion for Dor grouping.

#### 2.3.4 Group definitions

Group assignment in this study was based on the index operative record. The index procedure was defined as the primary sleeve gastrectomy that first met the inclusion criteria during the study period. Patients were divided into the Dor group and the non-Dor group according to whether concurrent Dor fundoplication was performed during the index procedure. The Dor group included patients who underwent sleeve gastrectomy with concurrent Dor fundoplication, regardless of whether they also underwent hiatal hernia repair. The non-Dor group included patients who underwent sleeve gastrectomy without Dor fundoplication; this group could include patients who had pure sleeve gastrectomy or sleeve gastrectomy with hiatal hernia repair.

If a patient underwent reoperation during follow-up for reflux, stenosis, weight regain, or other reasons, group assignment remained based on whether concurrent Dor fundoplication was performed during the index procedure, and was not reclassified according to subsequent treatments. Hiatal hernia repair, surgical approach, surgeon identity, and surgical year were not used as grouping criteria. This grouping method ensured that Dor fundoplication was clearly defined as the primary surgical strategy exposure, and avoided misattributing concurrent hiatal hernia repair or postoperative reinterventions to index Dor exposure.

### 2.4 Variable collection and definitions

#### 2.4.1 Data collection principles

All study variables were collected using a prespecified data extraction form, with data sources including the metabolic and bariatric surgery registry database, electronic medical records, endoscopy reports, endoscopic images or videos, upper gastrointestinal contrast reports, oesophageal function test reports, operative records, and prescription systems. Two trained researchers independently extracted core variables and reviewed preoperative Hill grade, hiatal hernia size, and preoperative GERD status while blinded to postoperative reflux outcomes. If discrepancies existed between data sources, priority was given to original reports, reviewable imaging, and operative records; if still unresolved, a senior gastrointestinal surgeon or gastroenterologist made the final decision.

#### 2.4.2 Preoperative Hill grade

Preoperative Hill grade was determined by retroflexed endoscopic view of the gastroesophageal flap valve, and endoscopy was required to have been performed within 12 months before surgery. In the primary analysis, Hill grade I–II was defined as low-risk Hill grade, and Hill grade III–IV as high-risk Hill grade. This threshold was chosen based on prior studies demonstrating that Hill grade III or IV is strongly associated with pathological acid exposure, severe reflux oesophagitis, and lower oesophageal sphincter dysfunction. These grades are characterised by a visible or absent gastroesophageal flap valve with hiatal herniation and loss of the angle of His, and have been shown to be independent predictors of postoperative GERD after sleeve gastrectomy. In secondary analyses, Hill grade was treated as an ordinal four-category variable (I, II, III, IV) to evaluate whether there was a gradient relationship between increasing Hill grade and the selection of Dor fundoplication. When original endoscopic images or videos were available, two researchers independently reviewed Hill grade; disagreements were adjudicated by a senior physician, and weighted kappa was used to assess agreement.

#### 2.4.3 Hiatal hernia size

Hiatal hernia size was recorded in centimetres, with the main source being the preoperative gastroscopy report. If the gastroscopy report did not clearly record size, the preoperative upper gastrointestinal contrast study, intraoperative measurements, or operative record description was used sequentially. Hiatal hernia size was treated both as a continuous variable and as a categorical variable, with categories defined as no hiatal hernia, small hiatal hernia ≤2 cm, and medium hiatal hernia >2 cm and ≤5 cm. Hernias >5 cm, paraoesophageal hernias, or those requiring complex hiatal repair were not included in the main analysis cohort. If the report described only “small hiatus hernia” or “mild hiatus hernia” without a specific measurement and the value could not be confirmed through other sources, it was not forcibly converted to centimetre values.

#### 2.4.4 GERD-related variables

Preoperative GERD symptoms were defined as the presence of heartburn, acid regurgitation, or reflux symptoms before the index surgery, as clearly documented by the clinician in outpatient or inpatient records. Preoperative PPI use was defined as regular proton pump inhibitor use for reflux-related symptoms for at least 4 weeks within 3 months before surgery. Reflux oesophagitis was recorded from the preoperative gastroscopy report and graded according to the Los Angeles classification. Los Angeles class A and B oesophagitis were recorded as baseline variables; Los Angeles class C or D oesophagitis were excluded. Barrett's oesophagus without dysplasia was recorded separately; Barrett's oesophagus with dysplasia was excluded.

#### 2.4.5 Other clinical and surgical variables

Baseline clinical variables collected included age, sex, baseline BMI, smoking status, diabetes, hypertension, obstructive sleep apnoea, American Society of Anesthesiologists grade, and previous abdominal surgery history. Baseline BMI was calculated using height and weight records closest to the index surgery date and within 30 days before surgery. Surgical variables included surgical approach, bougie size, whether hiatal hernia repair was performed, operative time, estimated blood loss, surgeon identity, and surgical year. All baseline covariates were defined as data formed before surgery or before the outcome occurred on the day of the index procedure; postoperative symptoms, medications, endoscopic findings, and complications were not included as baseline covariates in the primary adjustment models.

### 2.5 Outcome measures and follow-up

#### 2.5.1 Primary outcome measures

1. Performance of Dor fundoplication. Performance of Dor fundoplication was the primary decision outcome in this study, defined as whether concurrent Dor fundoplication was performed during the index sleeve gastrectomy. The index procedure was the primary sleeve gastrectomy that first met the inclusion criteria during the study period. Patients who underwent repeat anti-reflux surgery after the index procedure for reflux, stenosis, or other reasons did not change their index procedure grouping.
2. Treatment failure for reflux at 12 months postoperatively. Treatment failure for reflux at 12 months postoperatively was the key clinical outcome measure. The 12-month assessment window was defined as the follow-up record closest to 12 months within the period of 10 to 14 months after surgery. Treatment failure was defined as meeting any of the following: persistent or recurrent heartburn, acid regurgitation, or reflux symptoms requiring regular PPI use; GERD-Q score ≥8; postoperative gastroscopy showing Los Angeles class B or higher reflux oesophagitis; 24-hour pH monitoring showing acid exposure time >6% or DeMeester score >14.7; reintervention for refractory GERD; or conversion to Roux-en-Y gastric bypass for reflux-related reasons. Importantly, because this composite definition includes symptomatic and medication-based criteria, a patient could be classified as having treatment failure even without undergoing postoperative endoscopy or pH monitoring. This approach mitigates the bias that would arise from selectively performing objective tests only in symptomatic patients, as the primary outcome does not depend on the availability of these tests.

Regular PPI use was defined as continuous PPI use for reflux-related symptoms for ≥4 weeks within the 12-month assessment window, or a clear long-term PPI prescription record. Patients who did not undergo gastroscopy or pH monitoring were not considered negative for those tests; outcome status was determined solely on the basis of available symptom, medication, and clinical records.

#### 2.5.2 Secondary reflux-related outcome measures

1. *De novo* GERD. *De novo* GERD was defined as the appearance, within the 12-month assessment window, of typical reflux symptoms, regular PPI use, GERD-Q score ≥8, Los Angeles class B or higher reflux oesophagitis, or reintervention for reflux-related reasons, in patients who had no typical GERD symptoms, no regular PPI use, and no reflux oesophagitis before surgery.
2. Persistent GERD. Persistent GERD was defined as the persistence, within the 12-month assessment window, of typical reflux symptoms, regular PPI use, GERD-Q score ≥8, objective evidence of reflux, or reintervention for reflux-related reasons, in patients who had pre-existing GERD symptoms, regular PPI use, or reflux oesophagitis before surgery.
3. PPI discontinuation rate. PPI discontinuation was evaluated in patients who used PPIs regularly before surgery. PPI discontinuation was defined as no PPI use recorded for at least 8 weeks before the 12-month assessment window, and no re-prescription of PPIs for reflux symptoms. Short-term PPI use for postoperative gastric mucosal protection or for non-reflux indications was not counted as reflux-related PPI dependence.

#### 2.5.3 Safety, weight loss, and metabolic outcome measures

1. Safety outcomes. Safety outcomes included dysphagia, nausea or vomiting requiring intervention, sleeve stenosis, gastric leak, bleeding, 30-day readmission, 30-day reoperation, and Clavien-Dindo grade III or higher complications. Dysphagia was defined as swallowing restriction lasting more than 4 weeks, affecting eating, or requiring endoscopic or surgical intervention. Sleeve stenosis was defined as persistent food intolerance, vomiting, or dysphagia, with confirmation by gastroscopy or upper gastrointestinal contrast study of sleeve narrowing, twisting, or functional obstruction.
2. Weight-loss outcomes. Weight-loss outcomes included percentage of total weight loss and percentage of excess weight loss at 12 months postoperatively. Percentage of total weight loss was calculated as (preoperative weight − follow-up weight)/preoperative weight  × 100%. Percentage of excess weight loss was calculated as (preoperative weight − follow-up weight)/preoperative excess weight  × 100%. Postoperative weight was taken from the record closest to 12 months within the 12-month assessment window.
3. Diabetes remission. Diabetes remission was evaluated only in patients with preoperative type 2 diabetes. Complete remission was defined as glycated haemoglobin <6.0% and fasting glucose <5.6 mmol/L without glucose-lowering medication. Partial remission was defined as glycated haemoglobin 6.0%–6.4% or fasting glucose 5.6–6.9 mmol/L without glucose-lowering medication.

### 2.6 Statistical methods

All statistical analyses were performed using R 4.3.2 or Stata 18.0. Continuous variables were expressed as mean ± SD or median (IQR), and categorical variables as numbers (%). Baseline differences between groups were assessed using standardised mean differences, with SMD >0.10 indicating meaningful imbalance. Multivariable logistic regression, with robust standard errors clustered by surgeon, was used to identify factors associated with Dor selection, adjusting for age, sex, BMI, preoperative GERD symptoms, PPI use, reflux oesophagitis, hiatal hernia repair, surgical approach, year, and surgeon. To compare the relative contributions of Hill grade and hernia size, four sequential models (baseline, baseline + hernia size, baseline + Hill grade, and full) were evaluated using C-statistic, likelihood ratio test, AIC, Brier score, calibration curves, and decision curve analysis; nonlinearity was tested with restricted cubic splines and collinearity with VIF. Given non-randomised treatment assignment and the potential for selection bias, propensity score overlap weighting was applied to minimise confounding by indication, incorporating all baseline and surgical covariates, with balance assessed by SMD <0.10; This method assigns higher weights to patients with comparable propensity scores between groups, thereby creating a weighted population in which the distribution of measured covariates is balanced and the treatment effect estimate more closely approximates that from a randomised trial. The weighted generalised linear models, Cox regression, and linear/quantile regression were used for binary, time-to-event, and continuous outcomes, respectively. Missing baseline covariates (0–7.3%) were handled by multiple imputation (20 datasets) via chained equations, while treatment, exposures, and primary outcomes were not imputed. Prespecified sensitivity analyses included subgroup restrictions, alternative hernia size definitions, Hill grade as ordinal, inverse probability weighting, and complete gastroscopy follow-up. All tests were two-sided with *P* < 0.05 significant, and estimates are interpreted as associations, not causal effects, given the observational design.

## 3 Results

### 3.1 Patient screening, baseline characteristics, and data completeness

**Figure 1:**
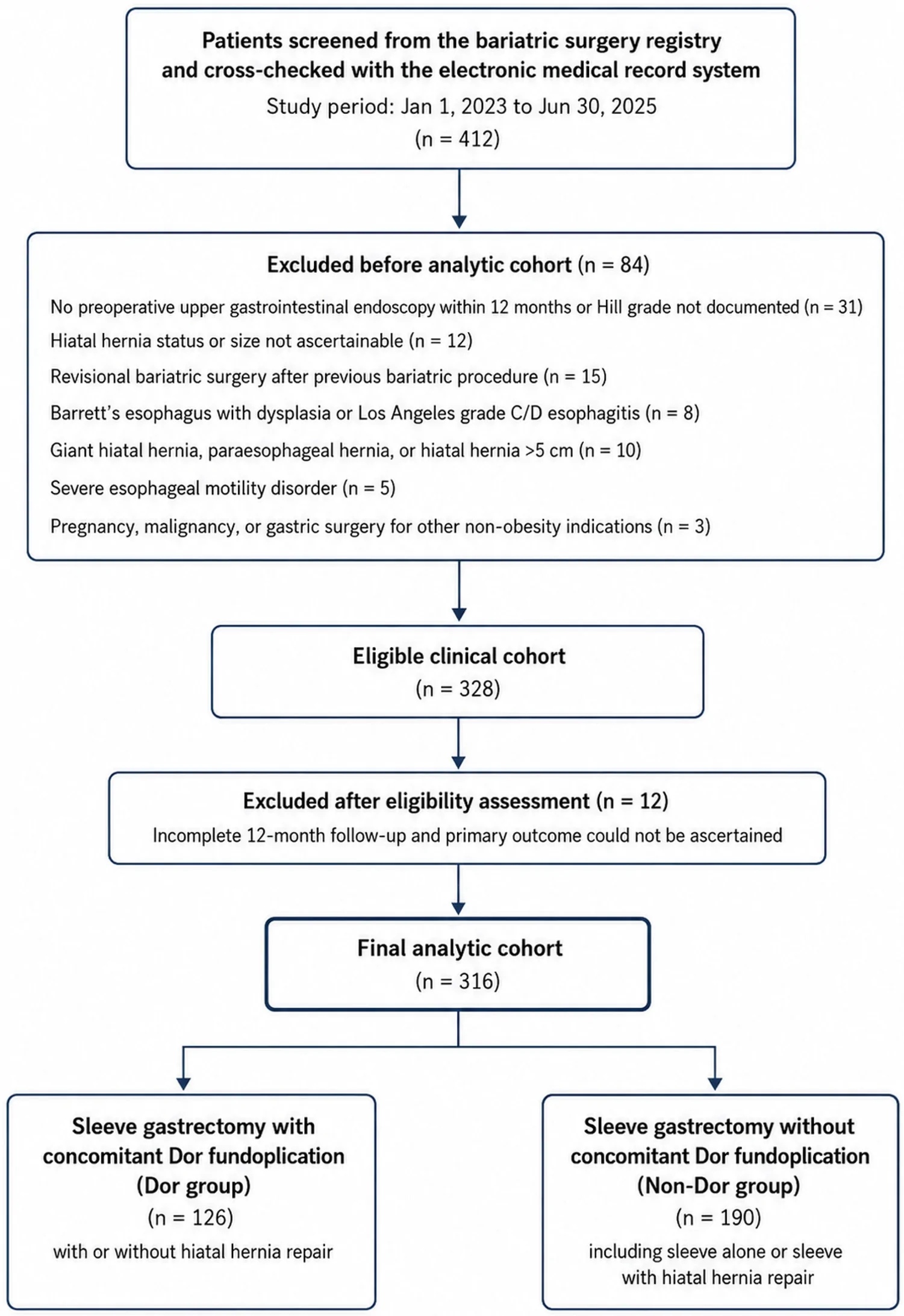
Patient screening flowchart.

Of 412 screened patients, 96 were excluded and 316 included (126 Dor, 190 non-Dor) (Figure 1). Both groups were similar in age, sex, BMI, smoking, type 2 diabetes, hypertension, OSA, ASA grade, and prior abdominal surgery (all *P* > 0.05, Table 1). The Dor group had higher preoperative reflux burden, including GERD symptoms (64.3% vs. 38.9%), regular PPI use (55.6% vs. 28.4%), GERD-Q score, GERD-HRQL score, and reflux oesophagitis (all *P* < 0.01). High-risk Hill grade (III–IV) was more common in the Dor group (69.0% vs. 31.1%, *P* < 0.001), hernia size was larger (2.4 vs. 1.3 cm, *P* < 0.001), and medium hernia proportion was higher (41.3% vs. 18.9%, *P* < 0.001). Concurrent hiatal hernia repair was more frequent in the Dor group (73.8% vs. 37.9%, *P* < 0.001). Hill grade agreement was good (weighted kappa 0.84). Surgical approach, bougie size, year, and surgeon did not differ between groups (all *P* > 0.05).

**Table 1:** Baseline characteristics, preoperative reflux risk, and surgery-related variables.

Variable	Overall	Dor group	Non-Dor group	Statistic	*P* value	SMD
	(*n* = 316)	(*n* = 126)	(*n* = 190)			
Demographic characteristics and baseline comorbidities						
Age, years	34.8 ± 9.4	35.1 ± 9.6	34.6 ± 9.3	t = 0.46	0.647	0.05
Female sex	217 (68.7)	88 (69.8)	129 (67.9)	*χ*^2^ = 0.13	0.715	0.04
BMI, kg/m^2^	38.3 ± 5.8	38.6 ± 5.9	38.1 ± 5.7	t = 0.75	0.455	0.09
Smoking status				χ^2^ = 0.35	0.84	0.08
Never smoker	221 (69.9)	90 (71.4)	131 (68.9)			
Former smoker	55 (17.4)	20 (15.9)	35 (18.4)			
Current smoker	40 (12.7)	16 (12.7)	24 (12.6)			
Type 2 diabetes	78 (24.7)	32 (25.4)	46 (24.2)	χ^2^ = 0.06	0.811	0.03
Hypertension	106 (33.5)	44 (34.9)	62 (32.6)	χ^2^ = 0.18	0.673	0.05
Obstructive sleep apnea	85 (26.9)	36 (28.6)	49 (25.8)	χ^2^ = 0.30	0.585	0.06
ASA class				χ^2^ = 0.18	0.675	0.05
Class II	205 (64.9)	80 (63.5)	125 (65.8)			
Class III–IV	111 (35.1)	46 (36.5)	65 (34.2)			
Previous abdominal surgery	60 (19.0)	24 (19.0)	36 (18.9)	χ^2^ = 0.00	0.982	0
Preoperative GERD-related variables						
Preoperative GERD symptoms	113 (35.8)	61 (48.4)	52 (27.4)	χ^2^ = 14.61	<0.001	0.44
Regular preoperative PPI use	86 (27.2)	50 (39.7)	36 (18.9)	χ^2^ = 16.44	<0.001	0.47
GERD-Q score, available *n* = 286	5.3 ± 3.5	6.6 ± 3.8	4.5 ± 3.1	t = 4.92	<0.001	0.62
GERD-HRQL score, available *n* = 238	9.5 ± 8.7	12.4 ± 9.2	7.6 ± 7.8	t = 4.19	<0.001	0.57
Reflux esophagitis, LA grade A/B	68 (21.5)	38 (30.2)	30 (15.8)	χ^2^ = 9.26	0.002	0.35
LA grade A	48 (15.2)	26 (20.6)	22 (11.6)			
LA grade B	20 (6.3)	12 (9.5)	8 (4.2)			
Barrett's esophagus without dysplasia	8 (2.5)	4 (3.2)	4 (2.1)	χ^2^ = 0.35	0.554	0.07
Gastritis	107 (33.9)	42 (33.3)	65 (34.2)	χ^2^ = 0.03	0.872	0.02
Helicobacter pylori positive	60 (19.0)	23 (18.3)	37 (19.5)	χ^2^ = 0.07	0.787	0.03
Hill grade and hiatal hernia variables						
Hill grade				χ^2^ = 38.24	<0.001	0.73
Hill grade I	59 (18.7)	11 (8.7)	48 (25.3)			
Hill grade II	126 (39.9)	37 (29.4)	89 (46.8)			
Hill grade III	91 (28.8)	53 (42.1)	38 (20.0)			
Hill grade IV	40 (12.7)	25 (19.8)	15 (7.9)			
High-risk Hill grade, III–IV	131 (41.5)	78 (61.9)	53 (27.9)	χ^2^ = 36.11	<0.001	0.73
Hiatal hernia size, cm	1.1 ± 1.3	1.6 ± 1.4	0.8 ± 1.2	t = 5.26	<0.001	0.62
Hiatal hernia category				χ^2^ = 32.33	<0.001	0.54
No hiatal hernia	145 (45.9)	36 (28.6)	109 (57.4)			
Small hiatal hernia, ≤2 cm	105 (33.2)	47 (37.3)	58 (30.5)			
Moderate hiatal hernia, >2 to ≤5 cm	66 (20.9)	43 (34.1)	23 (12.1)			
Preoperative ancillary tests and data completeness						
Upper gastrointestinal contrast study available	146 (46.2)	64 (50.8)	82 (43.2)	χ^2^ = 1.78	0.183	0.15
Esophageal manometry available	69 (21.8)	34 (27.0)	35 (18.4)	χ^2^ = 3.25	0.071	0.21
24-hour pH monitoring available	60 (19.0)	29 (23.0)	31 (16.3)	χ^2^ = 2.21	0.137	0.17
Pathologic acid exposure, among those with pH monitoring	26/60 (43.3)	15/29 (51.7)	11/31 (35.5)	χ^2^ = 1.61	0.205	0.33
Original endoscopic image or video available for review	248 (78.5)	100 (79.4)	148 (77.9)	χ^2^ = 0.10	0.755	0.04
Weighted kappa for Hill grade	0.82	—	—	—	—	—
Surgery-related variables						
Surgical approach				χ^2^ = 0.13	0.724	0.04
Laparoscopic	244 (77.2)	96 (76.2)	148 (77.9)			
Robotic-assisted	72 (22.8)	30 (23.8)	42 (22.1)			
Bougie size				χ^2^ = 0.22	0.64	0.05
36 Fr	244 (77.2)	99 (78.6)	145 (76.3)			
40 Fr	72 (22.8)	27 (21.4)	45 (23.7)			
Concomitant hiatal hernia repair	90 (28.5)	55 (43.7)	35 (18.4)	χ^2^ = 23.67	<0.001	0.56
Surgery year				χ^2^ = 1.17	0.557	0.12
2023	118 (37.3)	43 (34.1)	75 (39.5)			
2024	135 (42.7)	55 (43.7)	80 (42.1)			
Jan–Jun 2025	63 (19.9)	28 (22.2)	35 (18.4)			
Surgeon distribution				χ^2^ = 0.28	0.964	0.06
Surgeon A	93 (29.4)	38 (30.2)	55 (28.9)			
Surgeon B	80 (25.3)	31 (24.6)	49 (25.8)			
Surgeon C	76 (24.1)	29 (23.0)	47 (24.7)			
Surgeon D	67 (21.2)	28 (22.2)	39 (20.5)			

### 3.2 Factors associated with Dor fundoplication selection

**Figure 2:**
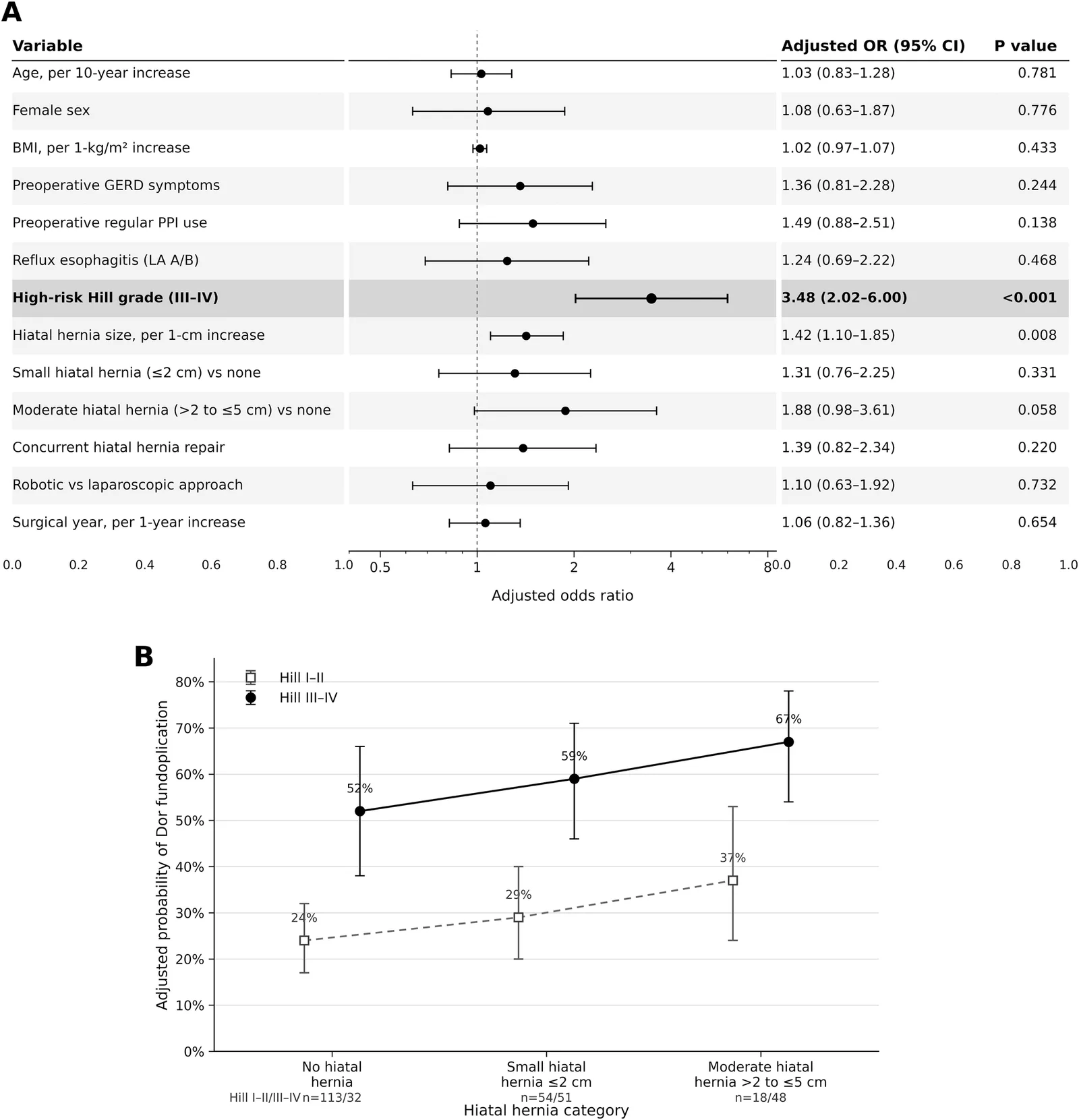
Factors associated with the selection of Dor fundoplication. **(A)** Forest plot from multivariable logistic regression showing the independent associations of preoperative clinical factors, reflux-related factors, Hill grade, hiatal hernia size, and surgical variables with the selection of Dor fundoplication. **(B)** Predicted probabilities of Dor fundoplication based on the adjusted model, stratified by Hill grade risk category and hiatal hernia size category, with error bars indicating 95% confidence intervals.

High-risk Hill grade was the strongest independent factor (adjusted OR 3.48, 95% CI 2.02–6.00, *P* < 0.001) (Figure 2A). Hernia size showed weaker association (adjusted OR 1.42 per cm, 95% CI 1.10–1.85, *P* = 0.008); small hernia was not significant (OR 1.31, 95% CI 0.76–2.25, *P* = 0.331), medium hernia showed a trend (OR 1.88, 95% CI 0.98–3.61, *P* = 0.058). Predicted probabilities of Dor selection were higher with Hill III–IV vs. I–II across no, small, and medium hernias: 52% vs. 24%, 59% vs. 29%, and 67% vs. 37%, respectively (Figure 2B). Preoperative GERD symptoms, PPI use, and reflux oesophagitis showed only weak positive associations.

### 3.3 Relative contributions of Hill grade and hernia size

**Figure 3:**
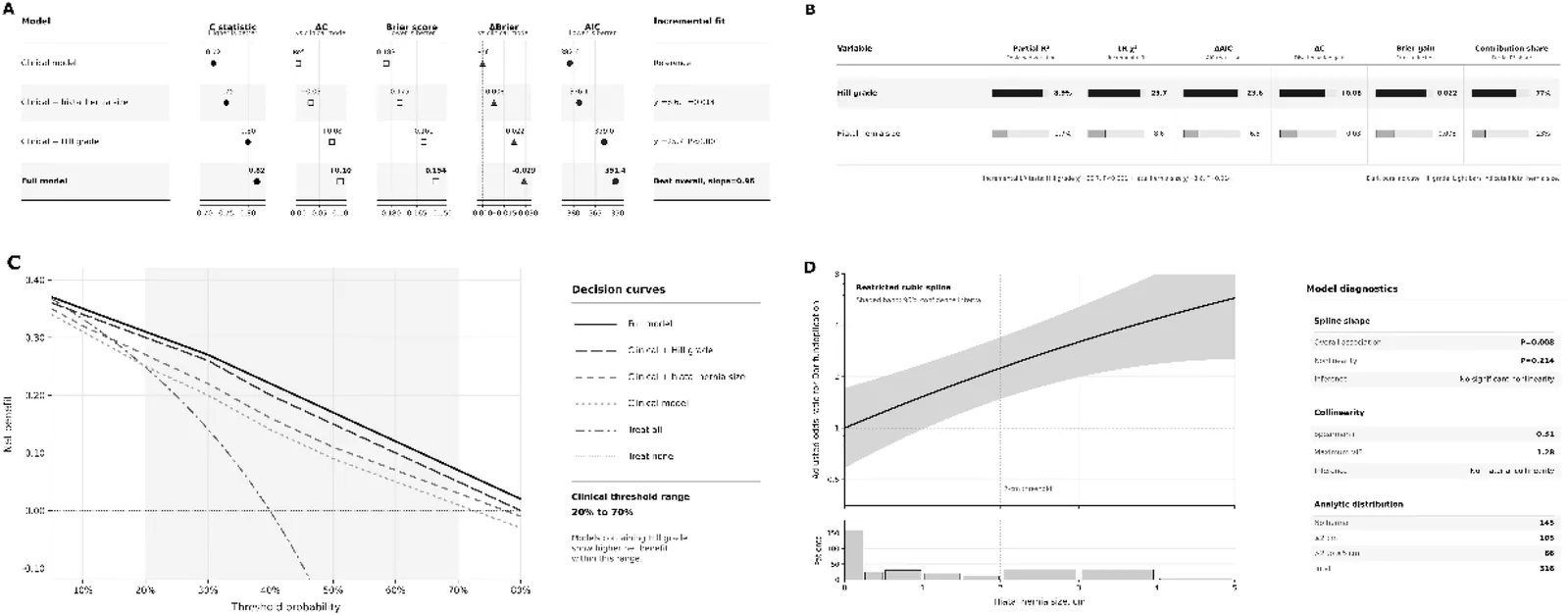
Incremental contributions of Hill grade and hiatal hernia size to the Dor decision model. **(A)** Comparison of sequential model performance, showing discrimination, calibration, and fit indices for the baseline clinical model, the model adding hiatal hernia size, the model adding Hill grade, and the full model. **(B)** Variable contribution analysis comparing Hill grade and hiatal hernia size in terms of partial R^2^, likelihood ratio *χ*^2^, AIC improvement, C-statistic increment, Brier score improvement, and relative contribution proportion. **(C)** Decision curve analysis evaluating the net benefit of different models across clinically relevant threshold probabilities. **(D)** Restricted cubic spline analysis of the relationship between hiatal hernia size and Dor selection, combined with collinearity diagnostics and variable distribution information to assess model stability.

Sequential models showed Hill grade provided greater incremental value than hernia size: adding hernia size increased C-statistic from 0.72 to 0.75, Brier from 0.183 to 0.175 (*χ*^2^ = 8.6, *P* = 0.014); adding Hill grade increased C-statistic to 0.80, Brier to 0.161 (*χ*^2^ = 25.7, *P* < 0.001). The full model performed best (C = 0.82, Brier = 0.154, lowest AIC, calibration slope = 0.96) (Figure 3A). Hill grade contributed higher partial *R*^2^ (8.9% vs. 2.7%) and proportion (77% vs. 23%) (Figure 3B). Decision curve analysis showed models with Hill grade had higher net benefit across 20%–70% thresholds; the full model performed best (Figure 3C). No nonlinearity for hernia size (*P* = 0.214); correlation modest (Spearman r = 0.31, VIF = 1.28) (Figure 3D).

### 3.4 Covariate balance after overlap weighting

**Figure 4:**
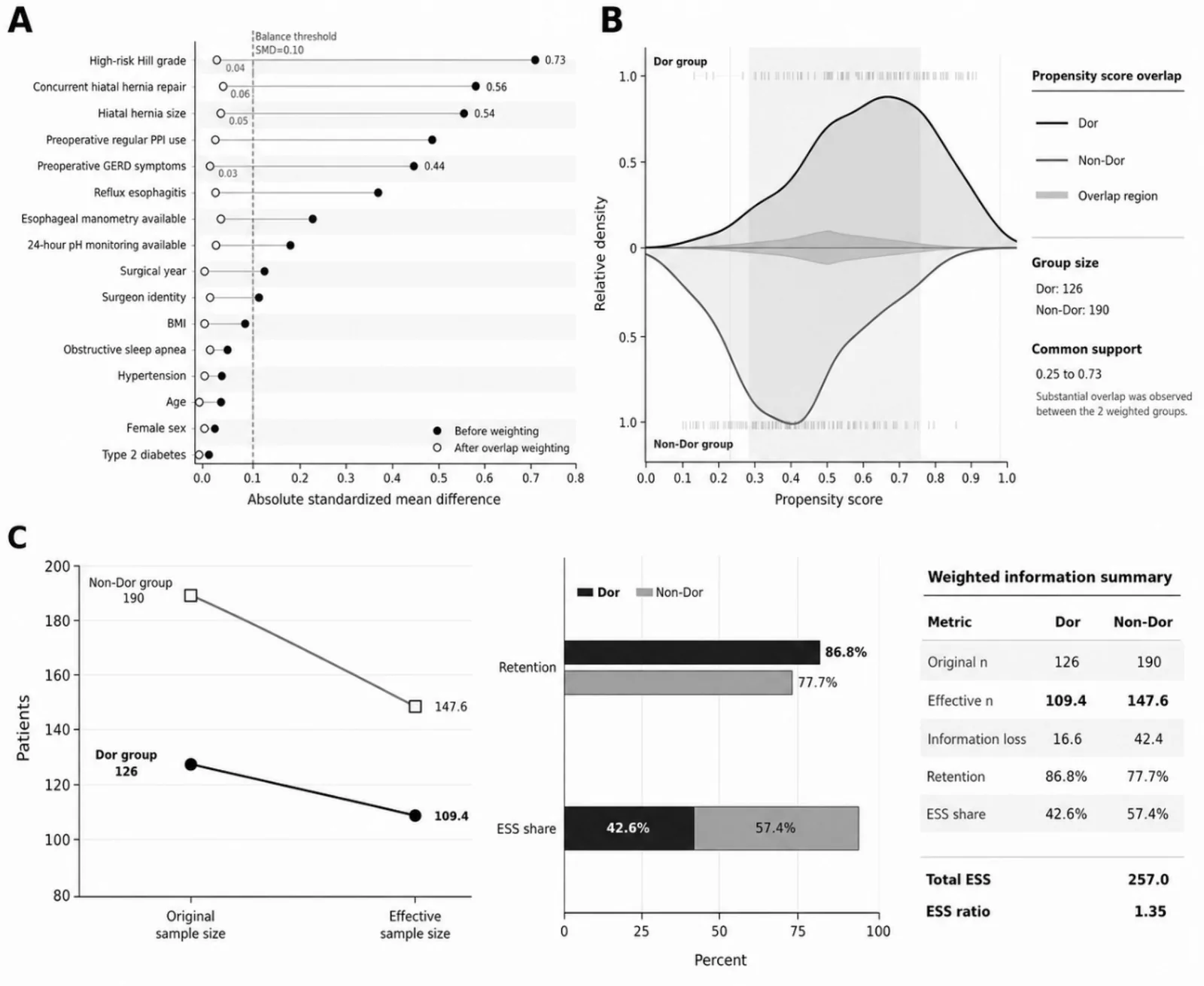
Covariate balance and effective information after propensity score overlap weighting. **(A)** Love plot showing standardised mean differences between the Dor and non-Dor groups before and after weighting, with the vertical reference line indicating the prespecified balance threshold of 0.10. **(B)** Propensity score distribution plot showing the overlap between the two groups within the common support region, used to assess comparability of the weighted populations. **(C)** Effective sample size analysis showing the original sample sizes, effective sample sizes after overlap weighting, information retention rates, and effective information proportions between groups, used to assess the stability of weighted estimates.

Before weighting, 8 covariates had SMD >0.10, with imbalances in high-risk Hill grade (0.73), hernia size (0.54), GERD symptoms (0.44), hiatal repair (0.56), PPI use (0.38), reflux oesophagitis (0.31), and BMI (0.15) (Figure 4A). After weighting, all SMDs <0.10 (Hill grade 0.04, hernia size 0.05, GERD 0.03, hiatal repair 0.06, PPI 0.04, oesophagitis 0.05, BMI 0.03) (Figure 4A). Propensity score overlap was adequate (common support 0.25–0.73) (Figure 4B). Effective sample sizes: Dor 109.4, non-Dor 147.6, total 257.0; retention rates 86.8% and 77.7% (Figure 4C).

### 3.5 Dor fundoplication and reflux outcomes

Treatment failure for reflux was lower in Dor vs. non-Dor (19.8% vs. 35.3%); after weighting: adjusted RD −12.8% (95% CI −22.1 to −3.5), RR 0.63 (95% CI 0.43–0.91), OR 0.51 (95% CI 0.30–0.87, *P* = 0.014) (Table 2). *De novo* GERD: 12.3% vs. 25.4%, RR 0.58 (95% CI 0.32–0.95); persistent GERD: 27.9% vs. 61.5%, RR 0.55 (95% CI 0.36–0.84); PPI discontinuation: 68.0% vs. 47.2%, RR 1.43 (95% CI 1.02–2.02) (Table 2). LA B+ oesophagitis: 11.3% vs. 26.2%, RR 0.54 (95% CI 0.29–0.98); pathological acid reflux: 20.0% vs. 44.2%, RR 0.57 (95% CI 0.26–1.13); conversion to RYGB: 0.8% vs. 3.7%, HR 0.32 (95% CI 0.04–1.55) (Table 2).

**Table 2:** Association of Dor fundoplication with reflux-related symptomatic, pharmacologic, endoscopic, physiologic, and reintervention outcomes at 12 months.

Outcome	Analysis population	Dor group	Non-Dor group	Overlap-weighted effect estimate	95% CI	*P* value
Primary reflux outcome						
Postoperative reflux treatment failure at 12 months	Full cohort, *n* = 316	25/126 (19.8%)	67/190 (35.3%)	RD −12.8%	−22.1% to −3.5%	0.014
				RR 0.63	0.43–0.91	0.014
				OR 0.51	0.30–0.87	0.014
Symptom-related outcomes						
Persistent or recurrent heartburn, acid regurgitation, or reflux	Full cohort, *n* = 316	20/126 (15.9%)	55/190 (28.9%)	RR 0.61	0.41–0.89	0.011
GERD-Q >= 8	Patients with available GERD-Q, *n* = 286	17/113 (15.0%)	49/173 (28.3%)	RR 0.57	0.36–0.90	0.016
GERD-HRQL score improvement >= 50%	Patients with preoperative GERD, *n* = 113	42/61 (68.9%)	24/52 (46.2%)	RR 1.48	1.07–2.06	0.018
Nocturnal reflux symptoms	Full cohort, *n* = 316	11/126 (8.7%)	35/190 (18.4%)	RR 0.53	0.29–0.94	0.03
New-onset GERD	Patients without preoperative GERD, *n* = 203	8/65 (12.3%)	35/138 (25.4%)	RR 0.58	0.32–0.95	0.032
Persistent GERD	Patients with preoperative GERD, *n* = 113	17/61 (27.9%)	32/52 (61.5%)	RR 0.55	0.36–0.84	0.006
Medication-related outcomes						
Regular PPI use at 12 months	Full cohort, *n* = 316	30/126 (23.8%)	77/190 (40.5%)	RR 0.66	0.47–0.91	0.012
PPI discontinuation at 12 months	Patients with preoperative regular PPI use, *n* = 86	34/50 (68.0%)	17/36 (47.2%)	RR 1.43	1.02–2.02	0.039
Need for add-on H2-receptor antagonist or prokinetic agent	Full cohort, *n* = 316	9/126 (7.1%)	31/190 (16.3%)	RR 0.50	0.26–0.92	0.027
Endoscopy-related outcomes						
Any LA-grade reflux esophagitis	Patients with available postoperative endoscopy, *n* = 174	16/71 (22.5%)	39/103 (37.9%)	RR 0.66	0.42–1.01	0.056
LA grade B or higher reflux esophagitis	Patients with available postoperative endoscopy, *n* = 174	8/71 (11.3%)	27/103 (26.2%)	RR 0.54	0.29–0.98	0.043
New-onset Barrett esophagus without dysplasia	Patients with available postoperative endoscopy, *n* = 174	1/71 (1.4%)	5/103 (4.9%)	RR 0.39	0.06–2.20	0.287
Physiologic reflux outcomes						
Pathologic acid reflux	Patients with available postoperative 24-hour pH monitoring, *n* = 68	5/25 (20.0%)	19/43 (44.2%)	RR 0.57	0.26–1.13	0.102
Acid exposure time >6%	Patients with available postoperative 24-hour pH monitoring, *n* = 68	5/25 (20.0%)	18/43 (41.9%)	RR 0.59	0.27–1.20	0.134
DeMeester score >14.7	Patients with available postoperative 24-hour pH monitoring, *n* = 68	6/25 (24.0%)	20/43 (46.5%)	RR 0.62	0.31–1.20	0.154
Reflux-related reintervention outcomes						
Emergency department visit or readmission due to GERD	Full cohort, *n* = 316	3/126 (2.4%)	13/190 (6.8%)	RR 0.42	0.13–1.21	0.105
Endoscopic or surgical reintervention due to GERD	Full cohort, *n* = 316	2/126 (1.6%)	10/190 (5.3%)	RR 0.36	0.08–1.28	0.116
Conversion to Roux-en-Y gastric bypass for reflux-related reasons	Full cohort, *n* = 316	1/126 (0.8%)	7/190 (3.7%)	HR 0.32	0.04–1.55	0.159

Postoperative gastroscopy was available in 174 patients (55.1%) and pH monitoring in 68 (21.5%). Analyses of these objective outcomes are restricted to the subgroups with available data and should be interpreted with caution, as patients with more severe symptoms may have been more likely to undergo these tests. This limitation underscores the value of our composite primary outcome, which does not require complete objective testing.

### 3.6 Safety, weight loss, and metabolic outcomes

Operative time longer with Dor: adjusted MD 23.5 min (95% CI 17.4–29.6, *P* < 0.001); hospital stay: 2.1 vs. 2.0 d, MD 0.1 (95% CI −0.2 to 0.4, *P* = 0.564) (Table 3). No increased risks: dysphagia 3.2% vs. 2.6%, RR 1.18 (95% CI 0.36–3.74); nausea/vomiting 5.6% vs. 4.7%, RR 1.09 (95% CI 0.43–2.72); Clavien-Dindo III+ 4.0% vs. 4.2%, RR 0.96 (95% CI 0.34–2.70). Low-frequency events (leak, bleeding, 30-d readmission, reoperation) did not differ (Table 3). At 12 months, %TWL: 29.4% vs. 30.7%, adjusted MD −1.1 (95% CI −2.5 to 0.3); %EWL: 72.8% vs. 75.1%, MD −2.0 (95% CI −6.1 to 2.1); diabetes remission: 70.0% vs. 72.9%, RR 0.97 (95% CI 0.72–1.29) (Table 3).

**Table 3:** Association of Dor fundoplication With safety, weight loss, and metabolic outcomes.

Outcome	Analysis population	Dor group	Non-Dor group	Weighted adjusted effect estimate	95% CI	*P* value
Perioperative measures						
Operative time, min	Full cohort, *n* = 316	112.4 ± 28.6	88.7 ± 24.9	MD 23.5	17.4–29.6	<0.001
Length of hospital stay, d	Full cohort, *n* = 316	2.1 (1.8–2.8)	2.0 (1.7–2.6)	MD 0.1	−0.2 to 0.4	0.564
Safety outcomes						
Clinically significant dysphagia	Full cohort, *n* = 316	4/126 (3.2%)	5/190 (2.6%)	RR 1.18	0.36–3.74	0.781
Nausea or vomiting requiring intervention	Full cohort, *n* = 316	7/126 (5.6%)	9/190 (4.7%)	RR 1.09	0.43–2.72	0.856
Sleeve stenosis	Full cohort, *n* = 316	2/126 (1.6%)	4/190 (2.1%)	RR 0.83	0.17–3.72	0.804
Gastric leak	Full cohort, *n* = 316	1/126 (0.8%)	2/190 (1.1%)	RR 0.78	0.08–6.83	0.821
Bleeding	Full cohort, *n* = 316	3/126 (2.4%)	5/190 (2.6%)	RR 0.93	0.24–3.40	0.914
30-day readmission	Full cohort, *n* = 316	6/126 (4.8%)	10/190 (5.3%)	RR 0.90	0.35–2.21	0.823
30-day reoperation	Full cohort, *n* = 316	2/126 (1.6%)	3/190 (1.6%)	RR 1.02	0.18–5.45	0.982
Clavien-Dindo grade III or higher complications	Full cohort, *n* = 316	5/126 (4.0%)	8/190 (4.2%)	RR 0.96	0.34–2.70	0.936
Weight loss outcomes						
Percentage total weight loss at 12 months, %TWL	Full cohort, *n* = 316	29.4 ± 6.7	30.7 ± 7.1	MD −1.1	−2.5 to 0.3	0.124
Percentage excess weight loss at 12 months, %EWL	Full cohort, *n* = 316	72.8 ± 17.9	75.1 ± 18.4	MD −2.0	−6.1 to 2.1	0.337
Metabolic outcomes						
Diabetes remission at 12 months	Patients with type 2 diabetes at baseline, *n* = 78	70.00%	72.90%	RR 0.97	0.72–1.29	0.814

### 3.7 Missing data and sensitivity analyses

**Figure 5:**
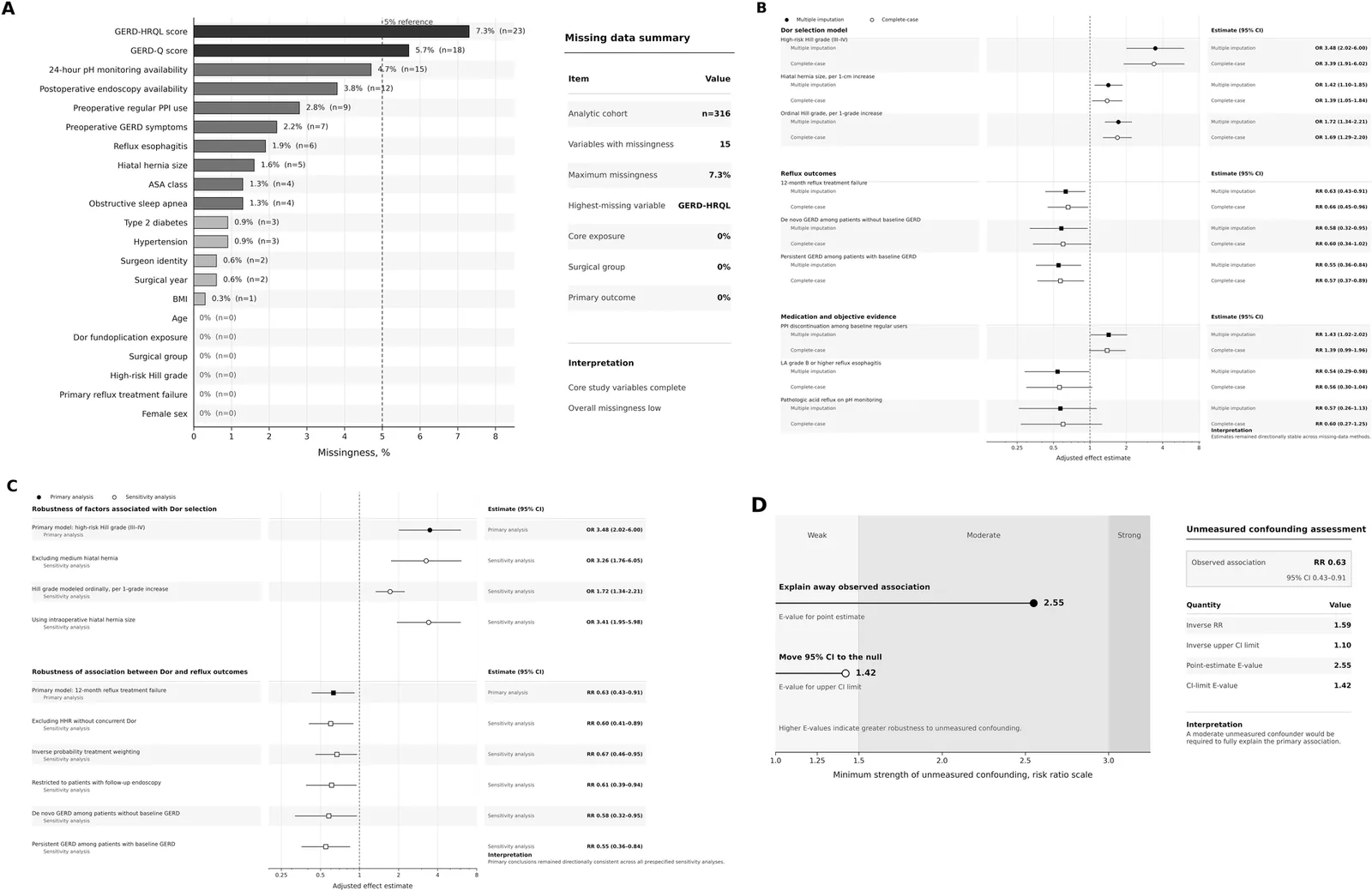
Missing data, sensitivity analyses, and unmeasured confounding assessment. **(A)** Missing data overview showing missing rates and numbers for core exposure variables, surgical grouping, primary outcomes, and key baseline covariates, used to assess data completeness. **(B)** Forest plot comparing multiple imputation analysis and complete-case analysis, showing consistency of effect estimates for the Dor selection model, primary reflux outcome, symptom outcomes, medication outcomes, and objective reflux evidence under different missing data handling methods. **(C)** Prespecified sensitivity analysis forest plot, evaluating the robustness of Hill grade in relation to Dor selection, and the association of Dor fundoplication with postoperative reflux outcomes across different populations, variable definitions, and weighting methods. **(D)** E-value robustness scale showing the minimum strength of association that unmeasured confounders would need to have to explain the observed association between Dor fundoplication and the primary reflux outcome.

Core variables and primary outcomes had no missing data; baseline missing rates 0%–7.3% (GERD-HRQL 7.3%, GERD-Q 5.7%) (Figure 5A). Multiple imputation results were consistent with complete-case analysis (Figure 5B). Sensitivity analyses confirmed robustness: after excluding medium hernias, Hill OR 3.26 (95% CI 1.76–6.05); Hill as ordinal, OR per grade 1.72 (95% CI 1.34–2.21); intraoperative hernia size, OR 3.41 (95% CI 1.95–5.98) (Figure 5C). Dor-reflux association remained stable: excluding hiatal repair without Dor, RR 0.60 (95% CI 0.41–0.89); IPTW, RR 0.67 (95% CI 0.46–0.95); complete gastroscopy follow-up, RR 0.61 (95% CI 0.39–0.94) (Figure 5C). E-value for primary association was 2.55 (upper CI limit E-value 1.42) (Figure 5D).

## 4 Discussion

Sleeve gastrectomy has become one of the most widely used procedures in metabolic and bariatric surgery, but postoperative gastroesophageal reflux can still affect long-term benefits in some patients. Reflux after sleeve gastrectomy does not always present as obvious heartburn or acid regurgitation; some patients may have increased objective acid exposure without prominent subjective symptoms (27). This discordance between symptoms and objective findings makes preoperative risk assessment and postoperative outcome evaluation more complex. This study focused on the clinical decision of whether to perform concurrent Dor fundoplication during sleeve gastrectomy, and compared the influence of preoperative Hill grade and hiatal hernia size on that decision. The results showed that high-risk Hill grade was more strongly associated with the selection of Dor than was hiatal hernia size. Dor fundoplication was also associated with a lower risk of treatment failure for reflux at 12 months. These findings suggest that the decision to add an anti-reflux procedure during sleeve gastrectomy should not rely solely on hernia size, but should also take into account the overall morphology and functional status of the anti-reflux barrier at the gastroesophageal junction.

The main finding of this study is that high-risk Hill grade was the dominant independent factor associated with the selection of Dor fundoplication. The development of GERD after sleeve gastrectomy is related to changes in the pressure gradient across the gastroesophageal junction, high pressure in the gastric sleeve, alteration of the angle of His, and changes in oesophageal clearance. Postoperative high-resolution manometry and impedance-pH studies have shown that some patients may have abnormal acid or non-acid reflux even when symptoms are atypical (28, 29). This suggests that preoperative symptoms alone cannot fully explain postoperative GERD risk, and that gastroesophageal junction dysfunction may be closer to the pathophysiological basis of reflux. Hill grade is not merely a description of the endoscopic appearance of the flap valve; it also reflects the closure of the gastroesophageal flap valve, fundic wrapping, and crural support. Recent integrated analyses have shown that objective physiological parameters and symptoms are often discordant after sleeve gastrectomy, and that procedure selection should incorporate indicators that more directly reflect the anti-reflux barrier itself (30). Preoperative endoscopic information has also been shown to predict GERD risk after sleeve gastrectomy (31). Therefore, the value of this study is not only to show that Hill grade is associated with postoperative reflux, but also to suggest that Hill grade may influence the surgeon's judgment of the need for concurrent anti-reflux reconstruction.

Hiatal hernia size was also associated with the selection of Dor in this study, but its explanatory power was weaker than that of Hill grade. Preoperative manometry studies have shown that oesophageal motility abnormalities help identify patients at high risk of GERD after sleeve gastrectomy, suggesting that hernia size cannot fully replace functional risk assessment (32). Controlled studies and multi-ethnic cohort studies have also indicated that preoperative GERD risk should be assessed by combining symptoms, anatomical structures, and procedural factors, and that small sliding hernias may not have a stable relationship with postoperative reflux symptoms (33, 34). This is consistent with our finding that small hernia had limited influence on the Dor decision. However, hernia itself remains clinically significant. Long-term follow-up studies have shown that standardised hiatal management may reduce reflux recurrence and improve reflux symptoms in some patients (35, 36). Propensity score-matched studies have also shown that concurrent hiatal hernia repair may affect perioperative risk and the probability of postoperative endoscopic intervention (37). Thus, hiatal hernia size is more an indicator of the degree of hiatal defect, whereas Hill grade is more a composite indicator of anti-reflux barrier failure. The two provide different information and should not be used interchangeably.

Moreover, Dor fundoplication was associated with a lower risk of treatment failure for reflux at 12 months. The theoretical basis of sleeve gastrectomy combined with fundoplication is to reconstruct the anterior anti-reflux barrier at the gastroesophageal junction while preserving the restrictive structure of the sleeve. Existing studies have shown that sleeve gastrectomy combined with Nissen fundoplication can improve GERD symptoms and maintain weight loss, but complete or near-complete wraps may increase technical difficulty and postoperative functional discomfort (38, 39). Comparative studies and randomised studies have also shown that sleeve gastrectomy combined with fundoplication may improve reflux-related outcomes, but the benefit depends on preoperative risk stratification, surgical details, and follow-up duration (40, 41). This study did not use Dor as a routine addition; rather, it analysed how preoperative variables influenced the choice of Dor and whether Dor was associated with improved reflux outcomes in a comparable population. This design more closely reflects real clinical decision-making and avoids pushing all patients towards an added anti-reflux procedure. The Dor group had lower rates of *de novo* GERD, persistent GERD, and regular PPI use, and the directions of endoscopic and pH-monitoring results were generally consistent. Related studies suggest that both the quality of hiatal repair and the reconstruction of the gastroesophageal junction geometry may affect reflux control (42, 43). This study used an anterior partial wrap, which may provide anterior support while reducing functional resistance from complete circumferential wrapping. This may explain the reduction in treatment failure for reflux without a significant increase in dysphagia or stenosis.

In terms of safety, this study showed that Dor fundoplication mainly increased operative time, but did not significantly increase hospital stay, severe complications, reoperation, or weight-loss and metabolic penalties. Real-world studies have shown that concurrent hiatal hernia repair during sleeve gastrectomy may not increase mortality, but may be associated with increased risks of readmission, reoperation, and subsequent intervention (44, 45). Other studies have suggested that concurrent hiatal hernia repair is generally feasible in the short term, while large-database studies have indicated that the risks of added procedures are affected by patient selection, centre experience, and technical approach (46, 47). Therefore, the finding that Dor increased operative time without significantly increasing severe complications in this study may be related to the relatively strict patient selection, exclusion of large and paraoesophageal hernias, and standardised surgical workflow. In terms of weight loss, the Dor and non-Dor groups showed no significant differences in %TWL, %EWL, or diabetes remission at 12 months, which is consistent with the generally comparable weight-loss results reported in studies of fundoplication sleeve gastrectomy (48). This is of practical importance: an anti-reflux addition can be an acceptable individualised strategy only if it does not compromise the primary weight-loss and metabolic goals of sleeve gastrectomy.

This study used overlap weighting, complete-case analysis, multiple imputation, sensitivity analyses, and E-value assessment to enhance robustness. Overlap weighting focuses on the clinical equipoise population for whom both treatment strategies are feasible, and therefore provides estimates closer to the real surgical decision-making scenario than the full-sample average effect. The target trial emulation framework also emphasises that observational studies need to clearly define the time of inclusion, treatment definitions, outcome windows, and analysis populations to reduce time-related bias and confounding by indication (49, 50). This study fixed the index procedure grouping, restricted the sources of preoperative variables, and closed the follow-up window and database lock time, and overall design met the basic requirements of high-quality observational research. The E-value analysis showed that only unmeasured confounders of at least moderate strength, simultaneously affecting both Dor selection and treatment failure for reflux, could fully explain the main association (51). This provides additional support for the observational findings. Nevertheless, the results of this study should still be interpreted as associations, not as causal effects demonstrated by a randomised trial. The STROBE guidelines emphasise that observational studies should clearly report study design, sources of bias, missing data, and sensitivity analyses (52).

This study has several limitations that should be considered when interpreting the findings. First, it was a single-centre retrospective cohort study. The decision to perform Dor fundoplication was influenced by surgeon experience, patient preference, and centre-specific technical pathways. Therefore, the results require validation in multicentre settings before they can be generalised to broader clinical practice. External validation in independent cohorts with standardised preoperative evaluation and systematic follow-up is crucial to confirm the stability of these findings across different patient populations and surgical practices.

Second, postoperative gastroscopy, manometry, and pH monitoring were not performed systematically in all patients. This introduces a risk of selective testing bias for objective reflux outcomes, as patients with more severe symptoms were likely overrepresented in the tested subgroups. To minimise the impact of this bias on our primary conclusion, we used a composite definition of treatment failure that included symptomatic and medication-based criteria, which were available for all patients, rather than relying solely on objective tests. Nonetheless, the subgroup analyses of endoscopic and pH-monitoring data should be viewed as exploratory and hypothesis-generating. Future studies should establish unified endoscopic and functional follow-up protocols, as multicentre systematic endoscopic follow-up has shown that some patients after sleeve gastrectomy may have endoscopic lesions even with mild symptoms (53).

Third, the follow-up period was limited to 12 months. This timeframe, while appropriate for assessing early weight loss and short-term reflux control, is insufficient to determine the durability of the observed reflux benefit. It cannot rule out the possibility that the Dor group might develop late complications such as wrap failure, dysphagia, or sleeve dilation over time. Conversely, the non-Dor group might experience delayed resolution of reflux as weight loss stabilises. Longer-term surveillance is essential to evaluate outcomes such as Barrett's oesophagus, sleeve dilation, and late revisional surgery, which may gradually appear over several years, as suggested by long-term follow-up studies (54). Our findings should therefore be considered as proof-of-concept at one year, and continued follow-up is needed to fully assess the risk-benefit profile of the combined procedure.

Fourth, although we employed propensity score overlap weighting to adjust for measured confounders and used various statistical methods to control for confounding, the allocation to Dor fundoplication was not randomised but was based on surgeon discretion. This introduces the potential for confounding by indication, whereby patients at higher reflux risk were preferentially assigned to receive the additional procedure. We cannot entirely exclude the possibility of residual or unmeasured confounding. However, the E-value analysis suggested that unmeasured confounders would need to have at least a moderate association with both treatment assignment and outcome to fully explain the observed effect, which provides additional reassurance.

Future prospective multicentre studies with systematic collection of preoperative Hill grade, precise hernia measurements, sleeve morphology, manometry, and impedance-pH data are needed. Such studies should also compare the results with salvage strategies such as conversion to Roux-en-Y gastric bypass over the long term (55). Recent studies have shown that symptom improvement after conversion to gastric bypass for reflux after sleeve gastrectomy is not entirely consistent (56), which suggests that precise preoperative stratification and anti-reflux strategy selection at the index procedure may have greater clinical value than postoperative salvage. Prospective validation would provide the necessary evidence for incorporating Hill grade into formal risk-stratification tools and clinical guidelines.

Based on our findings, we propose a preliminary risk-stratified framework to guide the decision for concurrent Dor fundoplication during sleeve gastrectomy. For patients with high-risk Hill grade (III–IV), concurrent Dor fundoplication should be strongly considered, especially when accompanied by moderate hiatal hernia (2–5 cm), significant preoperative reflux symptoms, regular PPI use, or objective evidence of pathological acid reflux. In this group, the expected benefit in reducing treatment failure for reflux, approximately 13% absolute risk reduction in our study, likely outweighs the additional operative time of about 24 min and the low risk of added morbidity. For patients with low-risk Hill grade (I–II), the decision should be more individualised. A small hiatal hernia (≤2 cm) alone may not be sufficient to warrant the procedure. However, the presence of larger hernias (>2 cm), severe reflux symptoms, or positive pH-monitoring findings could shift the balance in favour of adding Dor fundoplication. This framework is hypothesis-generating and requires prospective validation before being incorporated into formal clinical guidelines. It emphasises that the decision should be based on a composite assessment of the anti-reflux barrier (Hill grade), anatomical defect (hernia size), and clinical presentation (symptoms, PPI use, objective testing), rather than on any single parameter.

Our findings carry several practical implications. First, they suggest that current preoperative endoscopic assessment should incorporate systematic, standardised reporting of Hill grade alongside hiatal hernia size. Second, they provide evidence that Dor fundoplication can be effectively targeted to patients with high-risk Hill grade, in whom the absolute risk reduction in treatment failure for reflux is clinically meaningful at approximately 13%. Third, they reassure that when targeted appropriately, the added procedure does not impose a significant safety or weight-loss penalty, making it an acceptable individualised strategy. For clinical guidelines, our results support the consideration of Hill grade as a key risk-stratification variable when recommending whether to add an anti-reflux procedure to sleeve gastrectomy. However, given the observational design, we do not advocate for universal adoption of Dor fundoplication. Rather, we advocate for risk-stratified, individualised decision-making that integrates Hill grade, hernia size, symptoms, and patient preferences. The routine reporting of Hill grade in preoperative endoscopy reports should be encouraged to facilitate this approach.

## 5 Conclusion

In patients undergoing primary sleeve gastrectomy, preoperative Hill grade drove the decision for concurrent Dor fundoplication more strongly than did hiatal hernia size. High-risk Hill grade was stably and independently associated with the selection of Dor fundoplication, and its incremental explanatory contribution to the decision model was substantially greater than that of hiatal hernia size. This finding suggests that the preoperative decision for Dor should not be based solely on hernia size as a single anatomical parameter, but should place greater emphasis on the overall morphological assessment of the anti-reflux barrier at the gastroesophageal junction.

After adjustment using propensity score overlap weighting, Dor fundoplication was associated with a lower risk of treatment failure for reflux at 12 months, with consistent directions observed for *de novo* GERD, persistent GERD, PPI use, and objective reflux evidence. Dor fundoplication mainly increased operative time, but no clear safety, weight-loss, or metabolic penalties were observed. Overall, this study suggests that preoperative Hill grade is more strongly associated with the decision to perform concurrent Dor fundoplication during sleeve gastrectomy than hiatal hernia size. After propensity score overlap weighting, Dor fundoplication was associated with a lower risk of treatment failure for reflux at 12 months, without apparent safety or weight-loss penalties.

These findings highlight the potential value of Hill grade as a reference indicator when considering Dor fundoplication in individual patients. Routine, systematic reporting of Hill grade in preoperative endoscopy may facilitate more physiologically informed decision-making. However, given the single-centre retrospective design, external validation in independent, preferably multicentre cohorts is needed before these findings can inform clinical guidelines.

## Data Availability

The raw data supporting the conclusions of this article will be made available by the authors, without undue reservation.
